# Association Between Lifetime Cadmium Intake and Urinary Cadmium Concentration Among Residents of the Jinzu River Basin Following Soil Remediation

**DOI:** 10.3390/toxics14090762

**Published:** 2026-08-26

**Authors:** Yu Fujiwara, Kazuhiro Nogawa, Masaru Sakurai, Yuuka Watanabe, Masao Ishizaki, Yasumitsu Ogra, Yu-ki Tanaka, Hirotaro Iwase, Kayo Tanaka, Teruhiko Kido, Hideaki Nakagawa, Yasushi Suwazono

**Affiliations:** 1 Department of Occupational and Environmental Medicine, Graduate School of Medicine, Chiba University, Chiba 260-8670, Japan; 23fd0506@student.gs.chiba-u.jp (Y.F.); suwa@faculty.chiba-u.jp (Y.S.); 2 Department of Social and Environmental Medicine, Kanazawa Medical University, Kahoku 920-0293, Japan; m-sakura@kanazawa-med.ac.jp (M.S.); issa1@kanazawa-med.ac.jp (M.I.); hnakagaw@kanazawa-med.ac.jp (H.N.); 3 Graduate School of Pharmaceutical Sciences, Chiba University, Chiba 260-8675, Japan; ogra@chiba-u.jp (Y.O.); yu-ki.tanaka@chiba-u.jp (Y.-k.T.); 4 Department of Legal Medicine, Graduate School of Medicine, Chiba University, Chiba 260-8670, Japan; iwase@faculty.chiba-u.jp (H.I.); kayo-t4711@chiba-u.jp (K.T.); 5 Faculty of Health Sciences, Institute of Medical Pharmaceutical and Health Sciences, Kanazawa University, Kanazawa 920-0942, Japan; tkido@staff.kanazawa-u.ac.jp

**Keywords:** lifetime cadmium intake, urinary cadmium, soil remediation

## Abstract

Cadmium (Cd), a widespread pollutant, is primarily absorbed via diet and smoking. Chronic Cd exposure has been associated with various adverse health effects, including renal tubular and skeletal damage. Urinary Cd concentration accurately reflects cumulative Cd exposure owing to its strong correlation with renal cortical Cd levels. This study evaluated the accuracy of estimated lifetime Cd intake by examining its correlation with urinary Cd levels after soil remediation, an aspect unreported even in the Kakehashi River Basin. A total of 565 men and 450 women were included. Lifetime Cd intake (g) was estimated using an established exposure assessment model that incorporated individual residential history, rice Cd concentrations, and dietary assumptions. Urinary Cd concentrations were determined using inductively coupled plasma mass spectrometry. Linear regression analyses were conducted between lifetime cadmium intake and urinary cadmium concentration. The obtained standardized regression coefficients were 0.49 for men and 0.54 for women (both *p* < 0.001), indicating strong associations. Despite possible exposure misclassification, these findings support the utility of lifetime cadmium intake as a useful surrogate indicator of cumulative cadmium exposure and provide further evidence for its application in risk assessment and the derivation of tolerable cadmium intake values.

## 1. Introduction

Cadmium (Cd) is a toxic heavy metal that is widely distributed in the environment as a consequence of both natural geological processes and anthropogenic activities. Because Cd is characterized by an exceptionally long biological half-life in humans, exposure occurring over many years can lead to substantial accumulation in target organs, particularly the kidneys. Consequently, Cd continues to represent a major environmental and public health concern despite considerable progress in pollution control and environmental remediation [1,2]. Health effects associated with Cd exposure have been reported globally, including in Japan, Thailand, China, the United Kingdom, Belgium, and Sweden [2,3,4,5,6,7]. Contemporary population-based biomonitoring programs, including NHANES, continue to track cadmium exposure as a major environmental health concern, highlighting the ongoing need for robust methods to evaluate long-term Cd exposure [8,9]. Epidemiological and toxicological studies have linked Cd exposure to renal dysfunction, skeletal disorders, reproductive effects, cardiovascular disease, and increased mortality. Among these health outcomes, renal tubular dysfunction is generally regarded as the most sensitive and well-established manifestation of chronic Cd toxicity. Once a critical body burden is reached, tubular reabsorption mechanisms become impaired, resulting in increased urinary excretion of low-molecular-weight proteins and other biomarkers of renal damage [1,2,10]. In addition to renal toxicity, Cd has also been implicated in reproductive toxicity. Recent reviews and experimental studies have reported that cadmium impairs reproductive health through multiple mechanisms, including oxidative stress, DNA damage, and endocrine disruption [11,12,13]. Recent metabolomics-based studies have identified candidate biomarkers associated with environmental Cd exposure, suggesting that biomonitoring approaches are evolving beyond conventional measurements such as urinary Cd concentration (U-Cd) [14].

Japan has played a pivotal role in elucidating the human health effects of environmental Cd exposure. The most prominent example is Itai-itai disease, first identified among residents of the Jinzu River Basin in Toyama Prefecture [1,15]. Characterized by severe osteomalacia, renal dysfunction, chronic pain, skeletal deformities, and anemia, it remains one of the most significant cases of environmentally induced disease in public health history [16,17]. Similar cases have been reported in other Cd-polluted regions of Japan [18]. Moreover, studies have indicated a correlation between urinary Cd concentration (U-Cd), a marker of body burden, and β_2_-microglobulin levels, an indicator of proximal tubular dysfunction, even among residents of non-contaminated areas [7,19]. Individuals exhibiting such dysfunction have been shown to have reduced life expectancy [20,21,22,23,24], with the severity of dysfunction correlating with shorter survival [20]. Given these findings, establishing tolerable Cd intake levels is urgently needed to mitigate health risks.

Numerous studies have indicated a close relationship between U-Cd and Cd concentrations in the renal cortex, supporting its use as an index of long-term accumulated exposure [1,2]. U-Cd serves as a reliable biomarker of chronic workplace exposure, reflecting cumulative body burden rather than acute daily fluctuations in airborne cadmium levels [25]. A recent review has highlighted that even current low-level environmental Cd exposure may contribute to chronic kidney disease in the general population [26]. To quantify the health risks associated with this burden, several studies have employed benchmark dose methodologies to establish U-Cd thresholds for renal dysfunction [7,27,28]. These efforts have helped to set reference values for environmental health assessments and support the development of regulatory standards intended to reduce the health effects of Cd [29,30,31]. However, it should be noted that biological monitoring alone is unable to reconstruct historical exposure patterns or quantify cumulative intake over an individual’s lifetime. As a result, complementary approaches that estimate long-term exposure are necessary when investigating dose–response relationships and determining tolerable intake levels. A formula for estimating lifetime Cd intake [32] was previously proposed, and significant associations between estimated intake and health outcomes have been reported [32,33,34,35,36,37]. A strong correlation between U-Cd and estimated lifetime intake has also been reported in Kakehashi River Basin [38,39]. This study aimed to evaluate the accuracy of estimated lifetime cadmium intake by examining its correlation with urinary cadmium concentrations following soil remediation, which remains un-reported in previous studies, including those conducted in the Kakehashi River Basin. Evaluating this lifetime Cd intake estimation model in this population strengthens the scientific basis for its application in environmental epidemiology. Furthermore, identifying a persistent association between estimated lifetime Cd intake and U-Cd supports the utility of this methodology for reconstructing cumulative Cd exposure and developing evidence-based strategies to protect public health from the long-term consequences of environmental Cd contamination.

## 2. Materials and Methods

### 2.1. Participants

This study targeted residents living in Cd-contaminated areas along the Jinzu River Basin in Toyama Prefecture. Prior to the commencement of participant recruitment, explanatory meetings were held in six communities within the designated study area, with the objective of describing the study’s objectives and procedures. Thereafter, written invitations were disseminated to households via local administrative organizations. Residents who consented to participate were enrolled in a health survey conducted in 2020.

A total of 2118 individuals (1105 men and 1013 women) were invited to participate, and 2011 residents (1051 men and 960 women) completed the survey, corresponding to a participation rate of 94.9%. Information obtained from the residential history questionnaire was used to identify participants who had lived in areas with historically high Cd contamination prior to the implementation of soil remediation programs. First, 44 participants (25 men and 19 women) with incomplete residential history data were excluded. Next, 952 participants (461 men and 491 women) who did not reside in these historically contaminated areas were excluded. Consequently, 1015 participants (565 men and 450 women) were identified as eligible for inclusion in the study.

Written informed consent was obtained from all participants before enrollment. The study was conducted in accordance with the principles of the Declaration of Helsinki and was approved by the Ethics Committee of Kanazawa University (No. 968-I) and the Ethics Committee of Kanazawa Medical University (Nos. I-484 and I-750).

### 2.2. Data Collection

All participants were provided with urine collection kits and self-administered questionnaires prior to the administration of the survey. Participants were instructed to collect their first morning urine sample and submit it to the local community center. Although the urine collection kits were standard medical-grade and lacked trace metal-free certification, the samples were immediately transferred to the laboratory at Kanazawa Medical University and aliquoted into trace metal-free containers. Potential contamination from the kits was therefore minimal. Following collection, urine specimens were pH-adjusted and stored frozen at −30 °C until laboratory analysis. U-Cd was determined using inductively coupled plasma mass spectrometry (ICP-MS; 8800 Triple Quadrupole ICP-MS, Agilent Technologies, Santa Clara, CA, USA), an analytical technique widely recognized for its high sensitivity and precision in trace metal quantification. Samples were digested by wet ashing using concentrated nitric acid, diluted with ultrapure water, and analyzed by ICP-MS. Quality control was ensured using a blank (ultrapure water) and certified reference material (SRM 2668, Toxic Elements in Frozen Human Urine, NIST, Gaithersburg, MD, USA).

Potential analytical interference arising from molybdenum oxides was carefully evaluated. Prior to the analysis of samples, calibration experiments were conducted using molybdenum standard solutions to ascertain oxide formation rates. Based on these experiments, a correction equation was generated and subsequently applied to all urine measurements. Molybdenum concentrations were measured simultaneously during ICP-MS analysis to estimate and correct for analytical interference. The limit of detection and limit of quantification for U-Cd were 0.04 and 0.13 µg/L, respectively.

Urinary creatinine (Cr) concentrations were measured enzymatically by an ISO 15189-accredited [40] clinical laboratory (BML Inc., Tokyo, Japan). To account for variation in urinary dilution among participants, Cd concentrations were normalized to Cr concentration and expressed as micrograms per gram Cr (μg/g Cr).

Participants completed a structured questionnaire, collecting demographic information, including age and sex, as well as a complete residential history from birth through to 2020. Information on pre-existing renal disease and medication use was not collected in the present survey. Residential mobility was particularly emphasized because estimating cumulative Cd exposure required accurately determining the duration of residence in contaminated and non-contaminated areas. Each residential location was classified as either contaminated or non-contaminated based on environmental monitoring and soil-remediation data. Finally, the cumulative duration of residence in contaminated areas was calculated for each participant.

### 2.3. Lifetime Cd Intake

From 1971 to 1976, the Toyama Prefectural Department of Health and Welfare conducted surveys of Cd levels in unpolished non-glutinous rice collected from the Jinzu River Basin and neighboring river systems [41]. A total of 2446 rice samples were collected from 88 hamlets. Details of the rice sampling and analytical methods are described elsewhere [41,42]. Lifetime Cd intake was estimated by integrating individual residential histories with area-specific environmental Cd data. Information on all residential locations and duration of residence from birth to 2020 was obtained through interviews. Each residential period was classified based on whether the participant lived in a Cd-polluted hamlet or non-polluted area, taking into account the timing of soil remediation in each polluted hamlet (periods after soil remediation were classified as non-polluted). Specifically, hamlets subject to soil remediation were classified as Cd-polluted. Under the Agricultural Land Soil Pollution Prevention Law, paddy fields were designated for remediation if Cd concentrations in harvested rice were expected to exceed the regulatory limit (initially 1.0 mg/kg in 1976, revised to 0.4 mg/kg in 2010) [43].

To calculate cumulative intake, we applied the exposure assessment model proposed by Nogawa et al. [32]. Total Cd intake (g) during residence in a Cd-polluted hamlet prior to soil remediation was calculated as: [(hamlet-specific Cd concentration (µg/g) × 333.5 g/day + 34 µg/day) × 365 days/year × years of residence in the polluted hamlet] × 10^−6^, where 333.5 g/day, 34 µg/day, and 10^−6^ represent the average daily rice intake in 1970 [44], Cd intake from non-rice foods [44], and the conversion factor from µg to g, respectively. Total Cd intake (g) during residence in non-polluted areas was calculated as: (50 µg/day × 365 days/year × years of residence in the non-polluted area) × 10^−6^, where 50 µg/day represents the average Cd intake in non-polluted regions of Japan [45]. Total Cd intakes for each residential period were summed to estimate the cumulative lifetime Cd intake (g) from birth up to 2020.

### 2.4. Statistical Analysis

The association between lifetime Cd intake and U-Cd was examined using linear regression analysis. Log_10_-transformed lifetime Cd intake was entered as the independent variable, and log_10_-transformed U-Cd was entered as the dependent variable. Regression coefficients, standardized regression coefficients, 95% confidence intervals (CIs), and *p*-values were calculated. Analyses were stratified by sex, given the reported sex differences in Cd toxicokinetics and body burden. We performed an additional multiple linear regression analysis including age and smoking status (smoker vs. non-smoker) as potential confounders. In addition, as a sensitivity analysis, participants were stratified into two age groups (<65 years and ≥65 years), and regression analyses were performed to examine the association between log_10_-transformed lifetime Cd intake and log_10_-transformed U-Cd within each subgroup. All statistical analyses were performed using SPSS software (version 22; IBM Corp., Armonk, NY, USA). *p*-values < 0.05 were considered statistically significant.

## 3. Results

Table 1 summarizes the characteristics of the participants. The final sample included 565 men and 450 women, with mean ages of 65.5 and 66.0 years, respectively. The average total duration of residence in Cd-polluted areas was 30.7 years for men and 24.8 years for women. Smokers accounted for 24.4% of men and 3.3% of women. The geometric mean U-Cd concentrations were 1.23 µg/g Cr for men and 2.02 µg/g Cr for women. The geometric mean lifetime Cd intake was 2.66 µg/g Cr for men and 2.02 µg/g Cr for women.

Table 2 presents the results of regression analysis using log-transformed lifetime Cd intake as the independent variable and log-transformed U-Cd concentration as the dependent variable, stratified by sex. In men, the regression coefficient for log_10_-transformed lifetime Cd intake was 0.71 (95% CI: 0.61–0.82), with a standardized regression coefficient of 0.49 (*p* < 0.001). In women, the corresponding regression coefficient was 0.80 (95% CI: 0.68–0.92), and the standardized regression coefficient was 0.54 (*p* < 0.001). An additional multiple linear regression analysis was performed including age and smoking status as covariates. A stepwise variable selection procedure was applied, and smoking status was excluded from the final model. The association between log_10_-transformed lifetime Cd intake and log_10_-transformed U-Cd remained statistically significant after adjusting for age.

**Figure 1:**
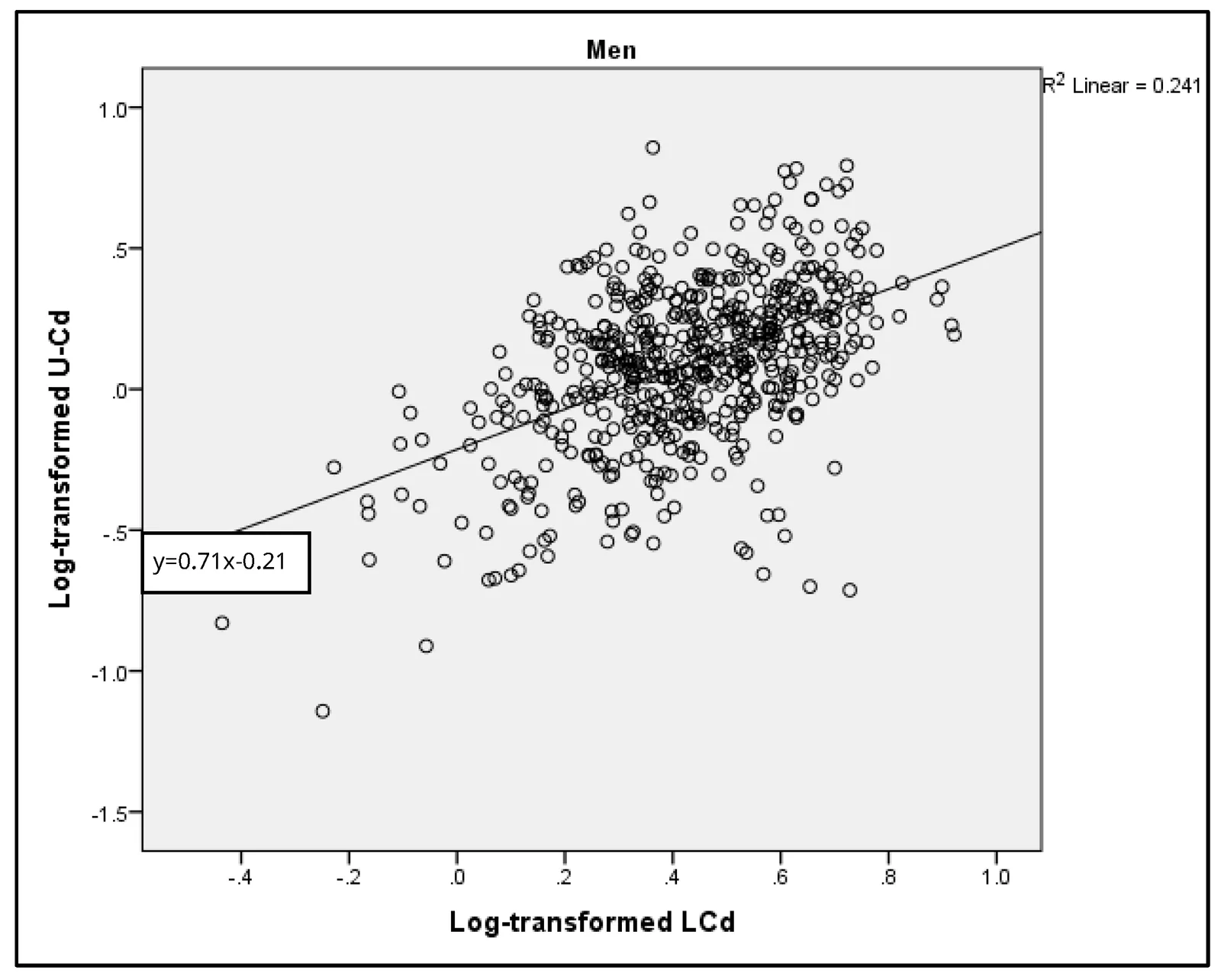
Scatter plot of log_10_-transformed lifetime Cd (LCd) intake and log_10_-transformed urinary U-Cd in men.

**Figure 2:**
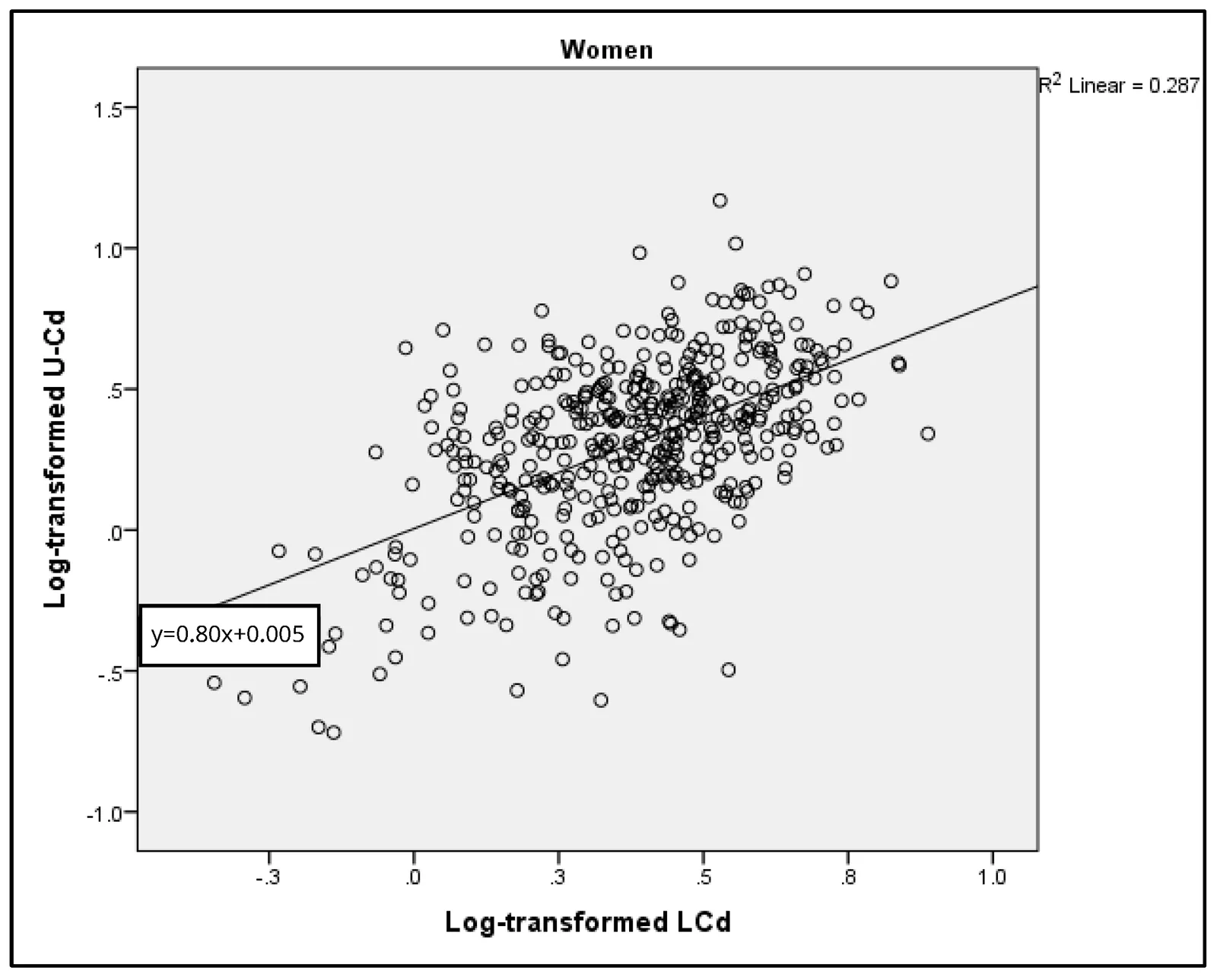
Scatter plot of log_10_-transformed lifetime Cd (LCd) intake and log_10_-transformed U-Cd in women.

As a sensitivity analysis, participants were stratified into two age groups (<65 years and ≥65 years), and regression analyses were performed to examine the association between log_10_-transformed Cd intake and log_10_-transformed U-Cd within each subgroup (Table 3). Significant associations were observed in both age groups. Scatter plots and regression lines for men and women are shown in Figure 1 and Figure 2, respectively, visually confirming the strong correlation.

## 4. Discussion

The present study found statistically significant and robust association between estimated lifetime Cd intake and U-Cd among residents of the Jinzu River Basin. The standardized regression coefficients were 0.49 in men and 0.54 in women, indicating that estimated lifetime Cd intake explained a substantial proportion of the variability in U-Cd. These findings provide further evidence supporting the adequacy of the lifetime Cd intake estimation model and suggest that the model remains applicable even in populations that have experienced considerable reductions in environmental Cd contamination following large-scale remediation activities. The magnitude of the standardized regression coefficients suggests a moderate-to-strong relationship between the exposure estimate and the biological marker of Cd body burden. Furthermore, the slightly larger standardized coefficient observed among women may imply a stronger correspondence between estimated lifetime Cd intake and urinary Cd concentration in females. The scatter plots revealed a positive correlation between urinary Cd concentrations and estimated lifetime Cd intake, irrespective of sex. However, individual variability was observed, reflecting differences in Cd absorption, metabolism, and excretion among participants. These findings indicate that the lifetime Cd intake model captures long-term cumulative exposure rather than only recent environmental conditions. Because urinary Cd is widely recognized as an indicator of body burden accumulated over decades [1,2,10], the observed associations suggest that lifetime Cd intake estimates reliably reflect biologically relevant exposure histories in populations affected by historical environmental contamination.

The observed association is biologically plausible because U-Cd reflects cumulative body burden accumulated over many years and is closely related to renal Cd concentration [1]. A particularly important aspect of our findings is that the study was conducted in a population whose environmental exposure conditions changed dramatically during their lifetime. Most previous studies examining relationships between cumulative Cd exposure and biological markers were performed in settings where environmental contamination remained relatively stable [32,33,34,35,36,37]. In contrast, residents of the Jinzu River Basin experienced substantial reductions in environmental Cd exposure following extensive soil remediation programs. Despite this environmental transition, estimated lifetime Cd intake remained strongly associated with U-Cd. This observation indicates that cumulative exposure reconstruction based on residential history and historical environmental measurements can produce adequate estimates even when exposure levels vary substantially over time.

Given that U-Cd has been linked to renal dysfunction and other adverse health outcomes in previous studies, accurate estimation of lifetime Cd exposure is crucial for risk assessment. Previously, we reported two studies on the relationship between U-Cd and lifetime Cd intake in residents of the Kakehashi River Basin. One study involved 100 men (mean age 45.8 years) and 98 women (mean age 50.7 years), with correlation coefficients of 0.88 for men and 0.84 for women [38]. The other study included 1419 men and 1745 women aged ≥50 years, with correlation coefficients of 0.61 and 0.59, respectively [39]. The present findings are consistent with these previous reports, despite differences in age distributions, study periods, and environmental conditions. Notably, our study indicates that the association remains evident even in a population that underwent substantial reductions in environmental Cd exposure following remediation efforts.

The major strengths of this study include a large sample size, the availability of geographically detailed historical rice-Cd data, and the unique opportunity to evaluate the exposure model in a population that underwent marked environmental remediation.

In the present study, U-Cd was corrected based on urinary Cr to account for variations in urine dilution. Although urine specific gravity adjustment is an alternative approach, recent evidence suggests that both Cr and specific gravity adjustments remain practical methods for dilution adjustment of urinary metal concentrations [46]. This approach was selected because it is widely applied in Cd biomonitoring research and facilitates direct comparison with previous studies.

One limitation of this study is the lack of data on blood parameters and detailed dietary habits and smoking intensity (e.g., pack-years). Consequently, renal function indicators, such as estimated glomerular filtration rate, could not be assessed, and these factors could not be controlled for as potential confounders in the analysis. Additionally, given that renal dysfunction may alter urinary Cd excretion, the possibility of reverse causation cannot be fully ruled out. Regarding occupational and other exposure sources, the study area is predominantly agricultural, and environmental Cd contamination has historically been attributed to emissions from the upstream mine. No other major industrial sources of Cd exposure, such as battery manufacturing plants or metal-processing facilities, are present in the area. Therefore, it is unlikely that occupational or alternative environmental Cd exposures substantially confounded the observed association.

Furthermore, the estimation of cumulative Cd exposure was based on Cd intake from rice, calculated from participants’ residential histories in contaminated areas. Although the estimation model incorporates detailed environmental and residential data, assumptions regarding individual rice consumption and dietary Cd intake were applied uniformly across participants. These factors may have introduced exposure misclassification due to individual variations. In particular, variations in daily rice intake, the use of non-local rice, year-to-year fluctuations in rice Cd levels, and recall inaccuracies regarding past residence could not be fully captured. Therefore, some non-differential exposure misclassification was likely present. Although more direct methods, such as duplicate-diet analyses and detailed food consumption surveys, can be used to estimate Cd intake, our approach may be less sensitive in capturing short-term fluctuations in exposure. However, given the long biological half-life of Cd and its tendency to accumulate in the body, accurately monitoring exposure over decades remains challenging with conventional dietary assessment methods. In this context, the estimation strategy employed in this study, which incorporates residential mobility and long-term environmental exposure, is considered appropriate for evaluating lifetime Cd burden. The strong correlation observed between lifetime Cd intake and urinary Cd concentrations further supports the reliability of this exposure assessment model. However, the performance of the model may vary in populations with lower exposure levels, distinct dietary patterns, or alternative Cd exposure sources. Therefore, further validation studies in diverse populations are warranted. We have previously investigated the relationship between lifetime Cd intake and health effects in the Kakehashi River Basin [32,33,34]. This study complements our earlier findings. The consistency of the findings with previous epidemiological observations and historical exposure patterns strengthens the credibility of the exposure assessment model. Future research should extend these findings by combining lifetime Cd intake estimates with benchmark dose methodologies and long-term health outcome data. Such investigations could help establish exposure-response relationships for renal dysfunction, mortality, osteoporosis, and other Cd-related health outcomes.

## 5. Conclusions

Our findings indicate a significant association between U-Cd and lifetime Cd intake, suggesting that the lifetime Cd intake model is useful for characterizing long-term Cd exposure when detailed environmental data are available. In future work, we aim to calculate Cd intake levels corresponding to tolerable U-Cd concentrations proposed by international agencies. Harmonizing tolerable intake values remains a challenge globally, and we believe this study contributes meaningfully to that effort. Furthermore, the persistence of the association despite extensive soil remediation indicates that historical exposure reconstruction can provide biologically meaningful information regarding cumulative Cd burden. This approach may be useful for future environmental risk assessments in populations affected by legacy contamination.

## Figures and Tables

**Table 1:** Characteristics of participants.

	Men (*n* = 565)		Women (*n* = 450)	
Variable	Mean	SD ^1^	Mean	SD ^1^
Age, years	65.5	13.3	66.0	14.2
Duration of residence in the polluted area, years	30.7	17.1	24.8	15.8
Smoking status (smokers vs. non-smokers)	%		%	
Smokers	24.4		3.3	
	GM ^2^	GSD ^3^	GM ^2^	GSD ^3^
U-Cd ^4^, µg/g Cr	1.23	1.96	2.02	2.03
Lifetime Cd ^5^ intake, g	2.66	1.59	2.38	1.61

^1^ Standard deviation. ^2^ Geometric mean. ^3^ Geometric standard deviation. ^4^ Cadmium. ^5^ Urinary cadmium.

**Table 2:** Regression coefficients for log_10_-transformed lifetime cadmium intake according to log_10_-transformed urinary cadmium.

U-Cd ^1^		Regression Coefficient (95% CI ^2^)	Standardized Regression Coefficient	*p*	R^2^
Men (*n* = 565)	Lifetime Cd ^3^ intake	0.71 (0.61, 0.82)	0.49	<0.001	0.24
Univariate model	Constant	−0.21 (−0.26, −0.16)		<0.001	
Age-adjusted	Lifetime Cd ^3^ intake	0.22 (0.09, 0.34)	0.15	0.001	0.40
model	Age, +10 years	0.11 (0.10, 0.13)	0.52	<0.001	
	Constant	−0.75 (−0.85, −0.66)		<0.001	
Women (*n* = 450)	Lifetime Cd ^3^ intake	0.80 (0.68, 0.92)	0.54	<0.001	0.29
Univariate model	Constant	0.005 (−0.05, 0.06)		0.846	
Age-adjusted	Lifetime Cd ^3^ intake	0.19 (0.05, 0.34)	0.13	0.010	0.45
model	Age, +10 years	0.12 (0.10, 0.15)	0.58	<0.001	
	Constant	−0.59 (−0.70, −0.48)		<0.001	

^1^ Urinary cadmium. ^2^ 95% confidence interval. ^3^ Cadmium. Smoking status was excluded by the stepwise model selection.

**Table 3:** Regression coefficients for log_10_-transformed lifetime cadmium intake according to log_10_-transformed urinary cadmium stratified into two age groups (<65 years and ≥65 years).

U-Cd ^1^		Regression Coefficient (95% CI ^2^)	Standardized Regression Coefficient	*p*
Men, <65 years	Lifetime Cd ^3^ intake	0.66 (0.49, 0.84)	0.45	<0.001
(*n* = 221)	Constant	−0.29 (−0.36, −0.23)		<0.001
≥65 years	Lifetime Cd ^3^ intake	0.29 (0.14, 0.44)	0.20	<0.001
(*n* = 344)	Constant	0.06 (−0.02, 0.14)		0.131
Women, <65 years	Lifetime Cd ^3^ intake	0.59 (0.35, 0.83)	0.35	<0.001
(*n* = 174)	Constant	−0.03 (−0.10, 0.04)		0.339
≥65 years	Lifetime Cd ^3^ intake	0.41 (0.25, 0.57)	0.29	<0.001
(*n* = 276)	Constant	0.24 (0.16, 0.32)		<0.001

^1^ Urinary cadmium. ^2^ 95% confidence interval. ^3^ Cadmium.

## Data Availability

The data presented in this study are available on request from the corresponding author due to privacy restrictions.

## Abbreviations

The following abbreviations are used in this manuscript:

Cd	Cadmium
Cr	Creatinine
ICP-MS	Inductively coupled plasma mass spectrometry
LCd	Lifetime Cd
U-Cd	Urinary Cd concentration
